# Simian malaria in wild macaques: first report from Hulu Selangor district, Selangor, Malaysia

**DOI:** 10.1186/s12936-015-0856-3

**Published:** 2015-10-05

**Authors:** Rumana Akter, Indra Vythilingam, Loke Tim Khaw, Rajes Qvist, Yvonne Ai-Lian Lim, Frankie Thomas Sitam, Balan Venugopalan, Shamala Devi Sekaran

**Affiliations:** Department of Medicine, Faculty of Medicine, University of Malaya, 50603 Kuala Lumpur, Malaysia; Department of Parasitology, Faculty of Medicine, University of Malaya, 50603 Kuala Lumpur, Malaysia; Department of Wildlife and National Park (PERHILITAN), KM10, Jalan Cheras, 56100 Kuala Lumpur, Malaysia; State Vector Borne Disease Control Unit, Selangor State Health Department, Selangor, Malaysia; Department of Microbiology, Faculty of Medicine, University of Malaya, 50603 Kuala Lumpur, Malaysia

**Keywords:** Simian malaria, Long-tailed macaques, Hulu Selangor

## Abstract

**Background:**

Malaria is a vector-borne parasitic disease which is prevalent in many developing countries. Recently, it has been found that *Plasmodium knowlesi*, a simian malaria parasite can be life-threatening to humans. Long-tailed macaques, which are widely distributed in Malaysia, are the natural hosts for simian malaria, including *P. knowlesi.* The aim of the present study was to determine the prevalence of simian malaria parasites in long-tailed macaques in the district of Hulu Selangor, Selangor, Malaysia.

**Methods:**

A total of 70 blood samples were collected from *Macaca fascicularis* dwelling in the forest of Hulu Selangor by the Department of Wildlife and National Parks Peninsular Malaysia, Kuala Lumpur, Malaysia. DNA was extracted using PureLink™ Genomic DNA Kits. Conventional and nested PCR were used to detect the genus and species of *Plasmodium* parasites respectively. In addition, phylogenetic analysis was carried out to confirm the species of *Plasmodium* parasites.

**Results:**

Thirty-five (50 %) of the 70 samples were positive for *Plasmodium* using genus-specific primers. These positive samples were then subjected to nested PCR targeting the 18S ribosomal RNA genes to detect all five simian malaria parasites: namely, *P. knowlesi, Plasmodium inui, Plasmodium cynomolgi, Plasmodium fieldi,* and *Plasmodium coatneyi.* All five species of simian malaria parasites were detected. Of these, *P. inui* was the predominant (65.7 %), followed by *P. knowlesi* (60 %), *P. cynomolgi* (51.4 %) *P. coatneyi* (45.7 %) and *P.fieldi* (2.9 %). A total of nine macaques had mono-infection with *P. knowlesi* (four)*, P. cynomolgi* (two), *P. coatneyi* (two) and *P. fieldi* (one). Eleven of the macaques had dual infections while 12 had triple infections. Three macaques were infected with four species of *Plasmodium*. Molecular and phylogenetic analysis confirmed the five species of *Plasmodium* parasites.

**Conclusion:**

This study has provided evidence to elucidate the presence of transmission of malaria parasites among the local macaques in Hulu Selangor. Since malaria is a zoonosis, it is important to determine the new control strategies for the control of malaria.

## Background

Malaria remains a public health problem in many tropical and sub-tropical regions. It is caused by the blood protozoan parasites of the genus *Plasmodium* which has been affecting humans since ancient times [1, 2]. According to the World Malaria Report 2014, around 198 million people were affected by malaria in 2013 with an estimated 58.4 million deaths [3]. Traditionally, only four species of *Plasmodium*, namely *Plasmodium falciparum*, *Plasmodium malariae, Plasmodium ovale*, and *Plasmodium vivax* were known to infect humans. However in 2004, Singh et al. [4] reported a large number of malaria cases in Sarawak to be caused by *Plasmodium knowlesi*, a simian malaria parasite. This resulted in the suggestion that *P*. *knowlesi* could be considered the fifth species of *Plasmodium* known to infect humans [5].

*Plasmodium knowlesi* mainly circulates among long-tailed (*Macaca fascicularis*) and pig-tailed macaques (*Macaca nemestrina*) [5]. In 1932, Knowles and Das Gupta reported the first experimental transmission of this simian malaria to human [6]. The first natural infection of *P.knowlesi* in human was reported later in 1965 in an American traveller who returned home from peninsular Malaysia [7].

Over the past decade, most countries in Southeast Asia and some in Asia have reported the presence of *P. knowlesi,* namely: Singapore [8], Thailand [9], Myanmar [10], Philippines [11], Indonesia [12, 13], Vietnam [14], Cambodia [15], Brunei [16], China [17]. Recently, Tyagi et al. reported human *P. knowlesi* infections from the Andaman and Nicobar Islands of India which are geographically nearby these Southeast Asian countries [18]. To date, 33 simian malarial parasites are documented in several prosimians, New World and Old World monkeys, African and Asian apes [19–22]. *Plasmodium knowlesi*, *Plasmodium cynomolgi*, *Plasmodium inui*, *Plasmodium coatneyi,* and *Plasmodium fieldi* are the five common species of simian malaria parasites in *M.fascicularis* and *M. nemestrina* [23].

Malaysia is considered endemic for malaria, mainly in the forested, mountainous and inaccessible areas of Malaysian Borneo comprising the states of Sabah and Sarawak, and peninsular Malaysia. A Malaria Eradication Programme was started in Sabah and Sarawak in 1961 and in peninsular Malaysia in 1967. Since then, there has been a significant reduction in the transmission of human malaria from *P*. *falciparum* and *P. vivax* whereas a significant increase in microscopy-diagnosed *P. knowlesi*/*P. malariae* cases was reported [24–26]. According to the Annual Report of the Ministry of Health Malaysia, *P*. *knowlesi* was the predominant (38 %) species followed by *P. vivax* (31 %) in 2012 [27]. Most of the cases were contributed by Sabah and Sarawak, while in peninsular Malaysia a high number of *P. knowlesi* malaria cases were reported from Pahang [28, 29]. The most developed state of Malaysia is Selangor which is still vulnerable to malaria [30]. In the last 5 years, *P*. *knowlesi*/*P*. *malariae* infection in humans was shown to be highest in Hulu Selangor district, one of the nine districts in Selangor [31]. This could be due to several factors, such as heightened human infringement into forests, high rate of construction and development, and the presence of secondary rainforests in the locality of urban and suburban residences which offer suitable habitats for mosquitoes and monkeys that can harbour various species of simian malaria parasites [30].

In recent years, an experimental analysis was carried out by Vythilingam et al. [31] to elucidate the transmission of *P. knowlesi* malaria in Hulu Selangor district. The study mainly focused on the epidemiology of *P. knowlesi* by determining the human infection and vector distribution. However, the limitation of the study was that there was no information regarding infection in the macaques from that same study area. In view of this gap of knowledge, it is imperative to understand the infection rate of simian malaria in macaque hosts. Hence, the main objective of this study was to determine the prevalence of malaria parasites in macaques, namely *P. knowlesi, P. cynomolgi, P.coatneyi, P. inui,* and *P. fieldi* in Hulu Selangor. The findings from this study have strengthened the evidence that Hulu Selangor is a potential risk area for knowlesi malaria transmission due to the presence of infected macaques. Besides, other malaria parasites such as *P. cynomolgi* and *P. inui* which have potential to infect humans were also present.

## Methods

### Collection of macaque blood samples

**Fig. 1:**
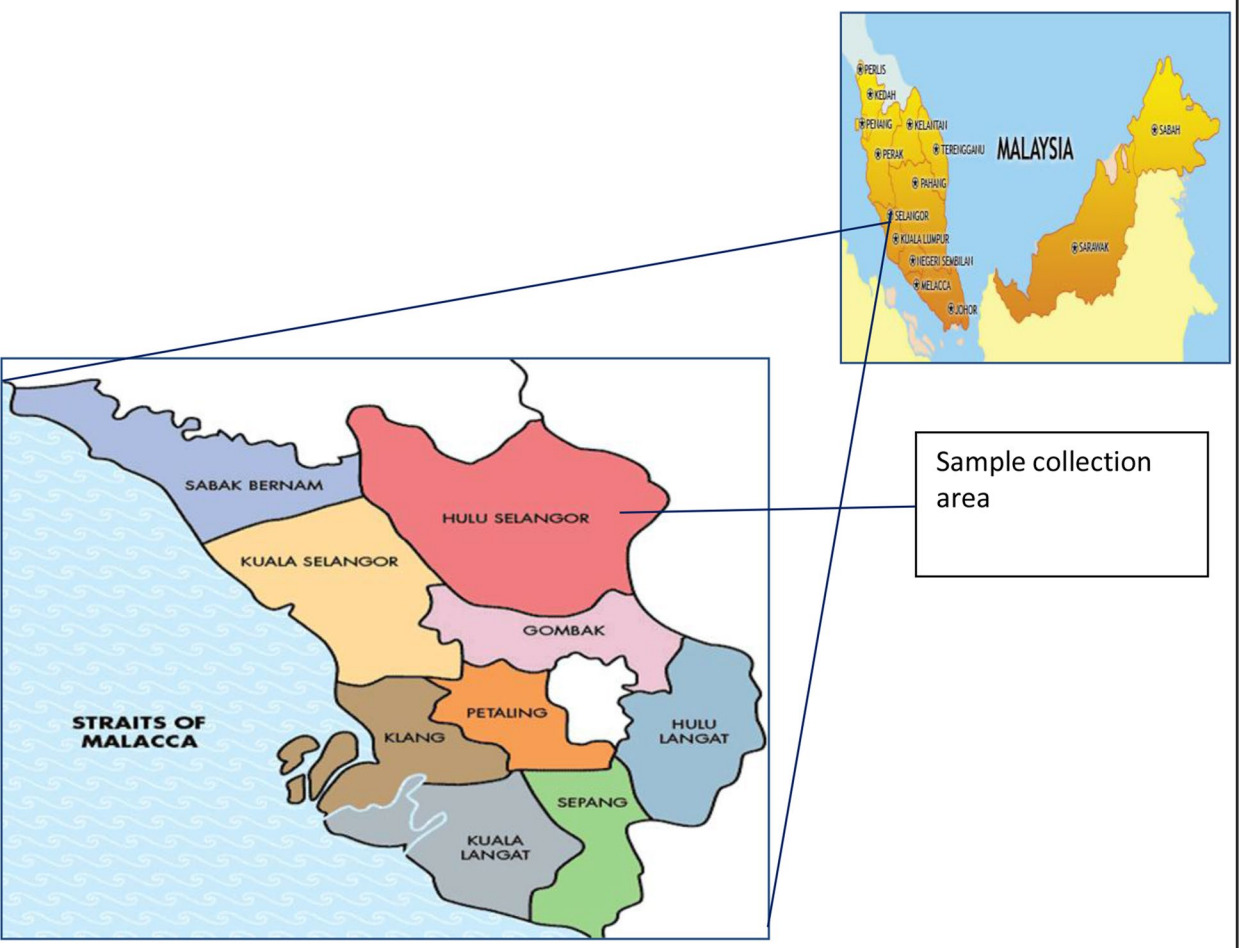
Map of Selangor State. Indicator showing the sample collection place of the study

In June 2014, a total of 70 macaque blood samples from Hulu Selangor (Fig. 1) were provided by the Department of Wildlife and National Parks Peninsular Malaysia, Kuala Lumpur, Malaysia. The macaques were caught by the Wildlife Department under a national surveillance programme, approved by the Department’s Animal Welfare Committee.

### DNA extraction

DNA was extracted from 200 µL blood using PureLink™ Genomic DNA Kit (Life Technologies, USA) according to manufacturer’s protocol. Purified DNA was eluted from the column with 100 μL elution buffer and stored at −20 °C until further use.

### Conventional PCR for screening of blood samples for detection of *Plasmodium* genus

Blood samples were screened for 18S ribosomal RNA genes for detection of *Plasmodium* by using conventional PCR with the thermal condition and primers sequences as described [32]. The PCR was carried out in a total of 20 µL volume containing 1× reaction buffer (5× Green Go Tag Flexi Buffer (Promega Madison WI, USA), 2.5 mM MgCl_2_ (Promega), 0.2 mM of each deoxynucleoside triphosphate (Promega), 0.3 µM of each primers (PlasF5′-AGT GTG TAT CAA TCG AGT TTC T-3′ and PlasR5′-CTT GTC ACT ACC TCT CTT CTT TAG A-3′) [32], 0.2 U of Go Taq DNA polymerase (Promega) and 2 µL genomic DNA template was used. Thermal conditions were as follows: an initial denaturation at 95 °C for 4 min followed by 35 cycles of amplification at 95 °C for 30 s, annealing at 55 °C for 30 s, extension at 72 °C for 30 s followed by a final extension at 72 °C for 2 min. Positive and negative controls were included with all the PCR reactions (Mega Cycler™, Edvotex, The Biotechnology and Education Company Ltd, China). The PCR products were subjected to electrophoresis in 2 % agarose gel and visualized under UV light.

### Nested PCR to detect *Plasmodium* species

All *Plasmodium* positive samples were subjected to nested PCR to detect the different species using the primer sequences as reported previously [4, 33, 34]. The primers were based on 18S ribosomal RNA and small sub-unit RNA genes. Nest 1 PCR mixtures were performed in a total of 20 µL reaction mixture. The amplification mixture contained the following in final concentration of 1× Green Go Tag Flexi Buffer (Promega) 2.5 mM MgCl_2_ (Promega), 200 mM each-dNTP (Promega), 0.25 µM of each primers, rPLU1: (5′-TCA AAG ATT AAG CCA TGC AAG TGA-3′ and rPLU5: (5′-CCT GTT GTT GCC TTA AAC TCC-3′) [34] and 1.25 U of Go Taq DNA polymerase (Promega) and 2 µL genomic DNA template was used for each reaction. The cycling parameters were as follows: an initial denaturation at 94 °C for 4 min followed by 35 cycles of denaturation at 94 °C for 30 s; annealing at 55 °C for 60 s, extension at 72 °C for 60 s followed by a final extension at 72 °C for 4 min. Two µL of nest 1 product was used as template DNA for nest 2 PCR. The PCR conditions were exactly the same as nest 1 except the annealing temperature. The primer sequence for the species and annealing temperatures are shown in Table 1. The PCR products were subjected to electrophoresis in 2 % agarose gel and visualized under UV light.

**Table 1:** Sequences of primers adopted from Singh et al. [4] and Lee et al. [34] with respective annealing temperatures and product size

Species	Name of primer	Sequences (5′–3′)	Annealing temperature (°C)	Product size
*P. knowlesi*	Pmk8	GTTAGCGAGAGCCACAAAAAAGCGAAT	60	153
	Pmk9r	ACTCAAAGTAACAAAATCTTCCGT		
*P. cynomolgi*	CY2F	GATTTGCTAAATTGCGGTCG	61	137
	CY4R	CGGTATGATAAGCCAGGGAAGT		
*P. coatneyi*	PctF1	CGCTTTTAGCTTAAATCCACATAACAGAC	61	504
	PctR1	GAGTCCTAACCCCGAAGGGAAAGG		
*P. inui*	PinF2	CGTATCGACTTTGTGGCATTTTTCTAC	61	479
	INAR3	GCAATCTAAGAGTTTTAACTCCTC		
*P. fieldi*	PfldF1	GGTCTTTTTTTTGCTTCGGTAATTA	62	421
	PfldR2	AGGCACTGAAGGAAGCAATCTAAGAGTTTC		

### Sequencing of PCR-amplified DNA

The PCR products were visualized over gel electrophoresis using 2 % agarose gel and the amplified products from the gel was purified and sequenced on an ABI 3730xl sequencer (Genomics Bioscience and Technology Co Ltd, Taiwan) using the PCR primers.

### Data analysis

The sequence data were analysed using the BLAST program [35] and the sequences aligned using the Clustal W programs in Bioedit package [36]. The phylogenetic trees were constructed by the neighbour-joining (NJ) and maximum likelihood (ML) methods with maximum composite likelihood model and Tamura 3-parameter model, respectively, with 1000 bootstrap replicates in MEGA 6.0 software [37–39].

## Results

**Fig. 2:**
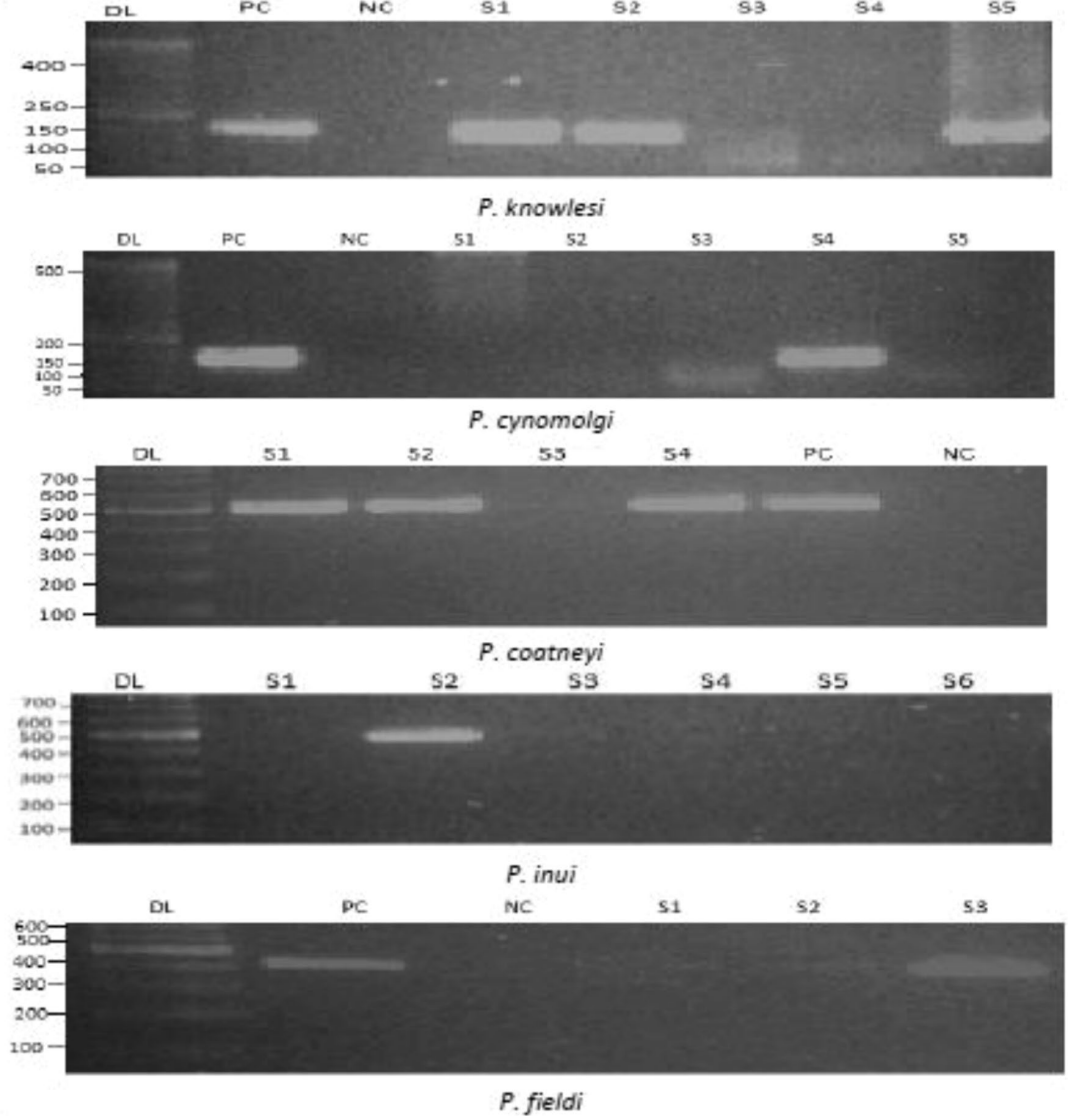
Molecular detection of simian malaria parasites in long-tailed macaques by nested PCR. The PCR products were subjected to electrophoresis in 2 % agarose gel. Here, the gel pictures show the positive and negative cases of four different species: *P. knowlesi*, *P. cynomolgi*, *P. coatneyi,* *P. inui and P. fieldi*. Here, DL indicates 50–100 bp ladder, PC and NC stands for positive and negative control. The expected product sizes for *P. knowlesi, P. cynomolgi, P. coatneyi, P. inui* and *P. fieldi* are 153, 137, 504, 479 and 421 bp

In this study, a total of 70 long-tailed macaques (*M. fascicularis*) blood samples were screened using conventional PCR to detect malaria parasites, among which 35 (50 %) samples were *Plasmodium* positive. From the positive samples, five species of *Plasmodium* parasites (Fig. 2) were detected. The infection rates in long-tailed macaques for *P. knowlesi, P. cynomolgi, P. coatneyi, P. inui* and *P. fieldi* were found to be 60, 51.4, 45.7, 65.7 and 2.9 % respectively (Table 2). Multiple infections with *Plasmodium* species were observed in these samples. The frequency of single, dual, triple, and quadruple infections of long-tailed macaques was 25.7, 31.4, 34.3, and 8.6 %, respectively (Table 3). The prevalence of mixed infection was higher (74.3 %) compared to mono-infection (25.7 %). Only nine macaques had mono-infection with *P. knowlesi* (four), *P. cynomolgi* (two) *P. coatneyi* (two) and *P. fieldi* (one). None of the macaques had mono-infection with *P. inui.* A total of 11 macaques (31.4 %) had dual infection, amongst them the prevalence of infection with *P. knowlesi* and *P. inui* was highest (36.4 %) followed by *P. knowlesi* and *P. cynomolgi* (9.1 %), *P. cynomolgi* and *P. inui* (27.3 %), *P. coatneyi* and *P. inui* (18.2 %), *P. cynomolgi* and *P. coatneyi* (9.1 %) (Table 3).

**Table 2:** Malaria infection in long tailed macaques

Species	Infected (N)	Percentage	Non infected (N)	Percentage	Total
*P. knowlesi*	21	60	14	40	35
*P. cynomolgi*	18	51.4	17	48.6	36
*P. coatneyi*	16	45.7	19	54.3	35
*P. inui*	23	65.7	12	34.3	35
*P. fieldi*	1	2.9	34	97.1	35

**Table 3:** Mixed species: infection in long tailed macaques

Infection type	Species	Infected	Total	Percentage
Single	Pk	4	9	25.7
	Pcy	2		
	Pct	2		
	Pfi	1		
Dual	Pk + Pin	4	11	31.4
	Pk + Pcy	1		
	Pcy + Pin	3		
	Pct + Pin	2		
	Pcy + Pct	1		
Triple	Pk + Pct + Pin	4	12	34.3
	Pk + Pcy + Pin	4		
	Pcy + Pct + Pin	3		
	Pk + Pcy + pct	1		
Quadruple	Pk + Pcy + Pct + Pin	3	3	8.6
Total		35	35	100

*Pk* *P. knowlesi*, *Pcy* *P. cynomolgi*, *Pct* *P. coatneyi*, *Pin* *P. inui*, *Pfi* *P. fieldi*

Among the total *Plasmodium*-positive cases, most of the macaques had triple infection (34.3 %) with *P. knowlesi*, *P. coatneyi* and *P. inui* (33.3 %), *P. knowlesi*, *P. cynomolgi* and *P. inui* (33.3 %), *P. cynomolgi, P. coatneyi* and *P. inui* (25 %), *P. knowlesi P. cynomolgi* and *P. coatneyi* (8.3 %) (Table 3). Whilst only three macaques were infected with all four species of *Plasmodium* parasites.

### Sequencing data analysis

**Fig. 3:**
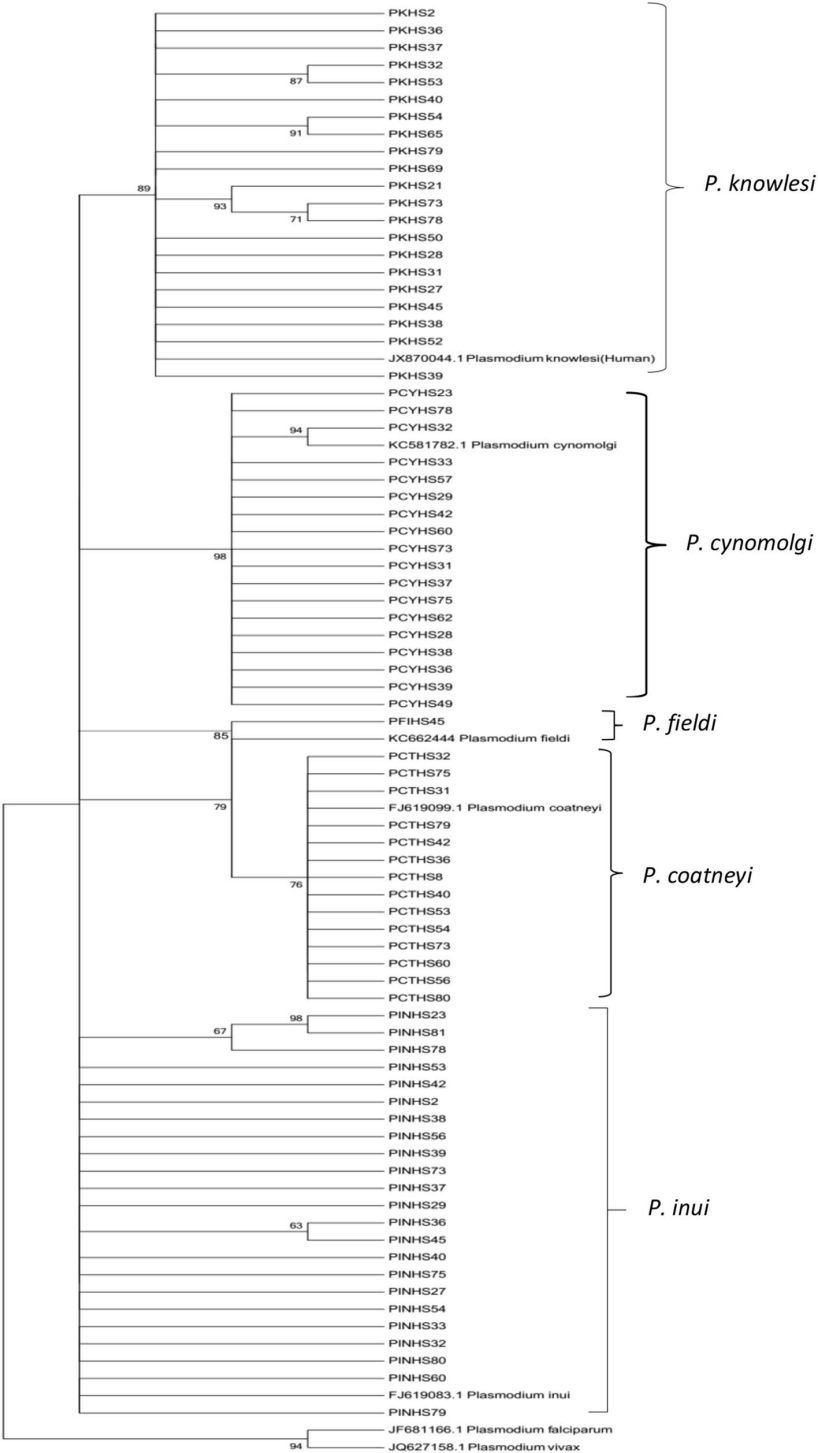
Phylogenetic tree of simian malaria parasites on the basis of 18S ribosomal RNA genes (A type) generated by neighbour-joining (NJ) method. NJ and ML methods yielded identical topologies and thus only the NJ tree is shown. The bootstraps percentage based on 1000 replicates are shown on the branches (bootstraps more than 60 % shown in branches)

The identified species-specific fragment of 18SSU rRNA of *Plasmodium* species was sequenced to confirm the species. The nucleotide sequence of 18SSU rRNA fragment of simian malaria parasites identified in this study was compared with the sequences obtained from GenBank. The phylogenetic reconstruction of the five simian malaria parasites using the NJ method showed (Fig. 3) that malaria parasites isolated from Hulu Selangor were clustered in five separate clades with the reference genes from GenBank. The topology originates using the ML method was similar to the NJ tree, which strongly supports the results obtained by NJ. Sequences reported in this study (18S rRNA A-type) have been submitted to GenBank, under accession numbers: KT124141-KT124216. The other sequences (also A-type) used as references obtained from GenBank are as follows: JX870044, KC581782, FJ619099, FJ619083, JF681166 and JQ627158.

## Discussion

The screening of macaques in Hulu Selangor illustrated that ~50 % of the macaques were positive and five common species of simian *Plasmodium*: *P. knowlesi*, *P. cynomolgi*, *P. coatneyi*, *P. inui* and *P. fieldi* were present. *Plasmodium inui* had the highest prevalence (65.7 %) followed by *P. knowlesi* (60 %). The high infection rate indicates a high intensity of transmission occurring among long-tailed macaques in Hulu Selangor. This is similar to a study conducted in Kapit, Sarawak where the prevalence of *P. inui* infection in long-tailed macaques (82 %) was predominant compared to other species [34]. In Sabah, Malaysian Borneo it was also found that *P. inui* was the most common parasite in both *M. fascicularis* and *M. nemestrina* [40]. However, in Singapore *P. knowlesi* was the predominant species (68.2 %) among long-tailed macaques [32]. In Kuala Lipis, Pahang, where macaques were screened only for *P. knowlesi*, 13.7 % were positive [29]. However, there was no *P. knowlesi* infection detected in Selangor from 41 macaques captured in urban areas and two macaques from Kuala Lumpur [29], which may be due to the absence of competent vectors for simian malaria transmission in urban areas surveyed [32]. It must be noted though, that the work did not report on other species of *Plasmodium.* In addition, Ho et al. reported that 64.5 % of 107 blood samples from long-tailed macaques were *Plasmodium* positive and among these 23.3 % were positive for *P*. *knowlesi* in Selangor [41].

In a study from Thailand, in 2008 they did not find any *P. knowlesi* in the macaques; only *P*. *inui* and *P. coatneyi* were found [42]. In a subsequent study, the infection rate of *P. inui* (38.9 %) was highest compared to *P. coatneyi* (16.7 %) followed by *P. knowlesi* at 5.6 % [43]. The high infection rate for *P*. *inui* is similar to the findings from the current study (65.7 %), although at a lower rate. Jones-Engel et al. reported one monkey (3.2 %) was infected by *P. knowlesi* among 31 in Indonesia [44]. It also needs to be noted that the study focused only on *P*. *knowlsei*. The results are summarized in Table 4.

**Table 4:** Summary of malaria infection in long tailed macaques in different countries

Study area	Pk	Pcy	Pin	Pct	Pfi	Year
Hulu Selangor (present study)	60 %	51.4 %	65.7 %	45.7 %	2.9 %	2015
Kapit, Sarawak Malaysian Borneo^a^	78 %	56 %	82 %	66 %	4 %	2011
Singapore^b^	68.2 %	66.6 %	1.5 %	3 %	16.7 %	2011
Kuala Lipis Pahang Malaysia^c^	13.7 %	–	–	–	–	2008
Kuala Lumpur and Selangor State Malaysia^c^	0 %	–	–	–	–	2008
Selangor State, Malaysia^d^	23.3 %	–	–	–	–	2010
Ulu Bernam, Kuala Kubu Bharu, Malaysia^e^	0 %	+	+	+	+	2013
Thailand^f^	0 %	–	+	+	–	2008
Thailand^g^	5.6 %	–	38.9 %	16.7 %	–	2010
Indonesia^h^	3.2 %	–	–	–	–	2007
Sabah, Malaysian Borneo^i^	+	+	+	+	+	2014

*Pk* *P. knowlesi*, *Pcy* *P. cynomolgi*, *Pin* *P. inui*, *Pct* *P. coatneyi*, *Pfi* *P. fieldi*, (+) positive

^a^Lee et al. [34]

^b^Irene [32]

^c^Vythilingam et al. [29]

^d^Ho et al. [41]

^e^Vythilingam et al. [31]

^f^Seethamchai et al. [42]

^g^Putaporntip et al. [43]

^h^Jones-Engel et al. [44],

^i^Muehlenbein et al. [40]

Simian malaria parasites are of great interest in Hulu Selangor as *P*. *knowlesi* infections are on the increase in Selangor. The recent emergence of *P*. *knowlesi* infections in humans may be due to close proximity of competent vectors, together with monkeys and humans, leading to conditions favourable for inter-species transmission to occur. This has occurred with deforestation and human encroachment on the natural habitats of the monkeys increasing the chance of human infection by simian malaria [31]. Recently, *Anophelesintrolatus* has been incriminated as a vector for *P.knowlesi* in Hulu Selangor [31]. The presence of this vector coupled with positive macaques shows that simian malaria can pose a serious public health problem to humans in the near future. It has also been reported that human *P. knowlesi* is an admixture of two divergent parasite populations associated with the two different macaque hosts dwelling in the forest [45] and studies have confirmed that there were two types of *P. knowlesi* parasites in patients from Sarawak [46]. In Sabah, it has been shown that there were differences in the sequences of *P.knowlesi* from long-tailed and pig-tailed macaques [40].

The zoonotic potential of *P. cynomolgi* parasite had been discovered after an accidental infection in humans by mosquito bites in the laboratory [47]. The prevalence of *P. cynomolgi* in long-tailed macaques was 51.4 %. In addition, this species has attracted a lot of attention recently as one case of natural infection in humans has been reported from Hulu Terengganu, Malaysia [48]. Besides, it has also been demonstrated that *P. inui* was infectious to humans in laboratory studies [49] and this was the predominant species (65.7 %) in this study. However, there has been no report of this parasite in humans and this may be due to misdiagnosis by microscopy since the parasites have overlapping morphological characteristics with human malaria parasites.

The findings of this study has demonstrated the prevalence of five simian malaria parasites in their natural host, further highlighting the need to deliberate new strategies to prevent the potential zoonosis of the other simian malarias. Efforts are ongoing to study the genetic diversity of these malaria parasites as recent studies have indicated their importance [45, 46]. Another prospective study would be the use of monkey-baited traps to capture and incriminate other mosquito species in their capacity to transmit simian malaria. The movement of these macaques also needs to be monitored in order to prevent their close proximity to humans.

## Conclusion

This study showed the prevalence of simian malarias in the local monkey population of Hulu Selangor. It found that four simian *Plasmodium* species were present in long-tailed macaques and occurred commonly as multiple infections. The study has provided evidence that *P. inui* was the most prevalent (65.7 %) malaria parasite followed by *P. knowlesi* in long-tailed macaques in Selangor. The significance of zoonotic simian malaria parasites cannot be overlooked in the assessment of accumulative interfaces between humans and wild animals in the development of urbanization. Potentially more samples should be collected in order to obtain a better understanding on the prevalence of simian malarias in monkeys all over Malaysia. These findings would be important for the planning and control of malaria strategies in the future. The genetic diversity of these parasites should also be studied.
